# Frequency of sodium channel genotypes and association with pyrethrum knockdown time in populations of Californian *Aedes aegypti*

**DOI:** 10.1186/s13071-021-04627-3

**Published:** 2021-03-06

**Authors:** Lindsey K. Mack, Erin Taylor Kelly, Yoosook Lee, Katherine K. Brisco, Kaiyuan Victoria Shen, Aamina Zahid, Tess van Schoor, Anthony J. Cornel, Geoffrey M. Attardo

**Affiliations:** 1 Department of Entomology and Nematology, College of Agriculture and Environmental Sciences, University of California, Davis, CA USA; 2 University of Florida-Florida Medical Entomology Laboratory, Vero Beach, FL USA; 3 Mosquito Control Research Laboratory, Kearney Agricultural Center, Department of Entomology and Nematology, University of California, Davis, CA USA

## Abstract

**Background:**

Since their detection in 2013, *Aedes aegypti* has become a widespread urban pest in California. The availability of cryptic larval breeding sites in residential areas and resistance to insecticides pose significant challenges to control efforts. Resistance to pyrethroids is largely attributed to mutations in the voltage gated sodium channels (VGSC), the pyrethroid site of action. However, past studies have indicated that VGSC mutations may not be entirely predictive of the observed resistance phenotype.

**Methods:**

To investigate the frequencies of VGSC mutations and the relationship with pyrethroid insecticide resistance in California, we sampled *Ae. aegypti* from four locations in the Central Valley, and the Greater Los Angeles area. Mosquitoes from each location were subjected to an individual pyrethrum bottle bioassay to determine knockdown times. A subset of assayed mosquitoes from each location was then analyzed to determine the composition of 5 single nucleotide polymorphism (SNP) loci within the VGSC gene.

**Results:**

The distribution of knockdown times for each of the five Californian populations sampled was non-parametric with potentially bimodal distributions. One group succumbs to insecticidal effects around 35–45 min and the second group lasts up to and beyond the termination of the assay (120+ min). We detected 5 polymorphic VGSC SNPs within the sampled California populations. One is potentially new and alternatively spliced (I915K), and four are documented and associated with resistance: F1534C, V1016I, V410L and S723T. The Central Valley populations (Clovis, Dinuba, Sanger and Kingsburg) are fairly homogenous with only 5% of the mosquitoes showing heterozygosity at any given position. In the Greater LA mosquitoes, 55% had at least one susceptible allele at any of the five SNP loci. The known resistance allele F1534C was detected in almost all sampled mosquitoes (99.4%). We also observe significant heterogeneity in the knockdown phenotypes of individuals with the identical VGSC haplotypes suggesting the presence of additional undefined resistance mechanisms.

**Conclusions:**

Resistance associated VGSC SNPs are prevalent, particularly in the Central Valley. Interestingly, among mosquitoes carrying all 4 resistance associated SNPs, we observe significant heterogeneity in bottle bioassay profiles suggesting that other mechanisms are important to the individual resistance of *Ae. aegypti* in California. Keywords: *Aedes aegypti*, Resistance, Pyrethroid, IPLEX genotyping, Voltage gated sodium channel, California.
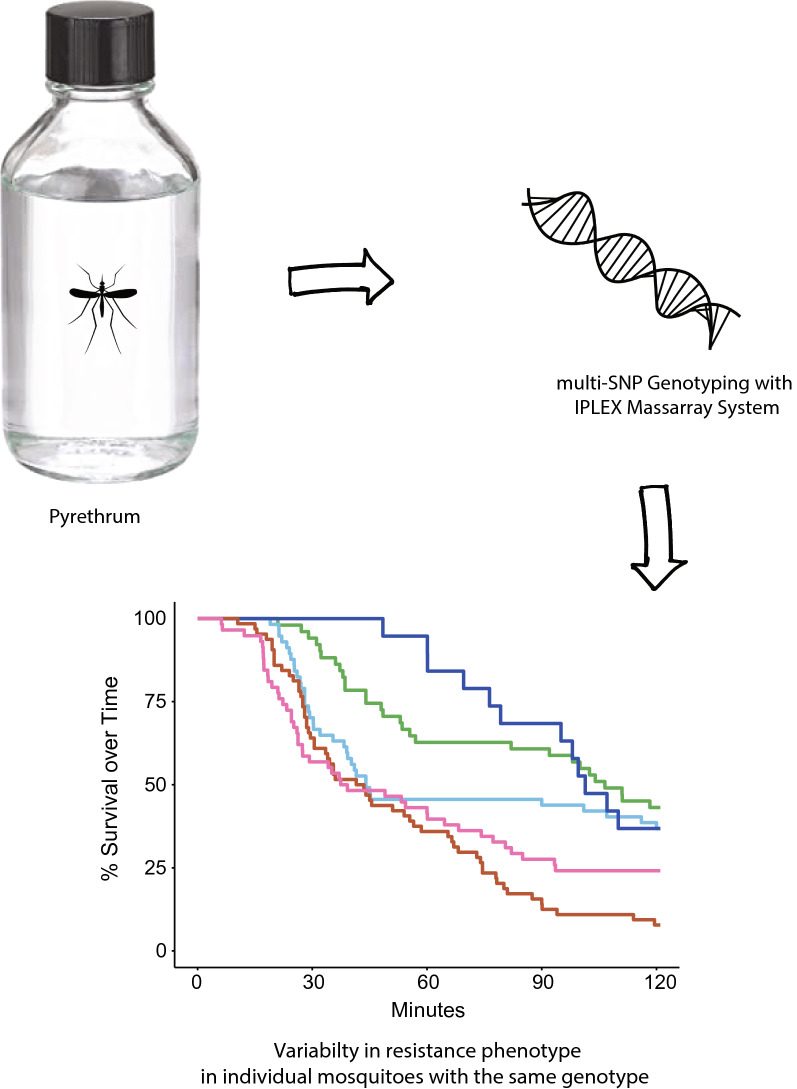

## Background

The yellow fever mosquito, *Aedes aegypti *(*Linnaeus 1762*), is a major vector of arboviruses such as dengue, Zika, chikungunya, and yellow fever viruses. This medically important vector was detected in California in 2013 in response to a residential service request [1]. It has now been detected in 17 counties throughout the state despite aggressive surveillance and treatment efforts, presenting a significant challenge to local control agencies [1–3].

Questions remain about the timing of *Aedes aegypti*’*s* arrival to California, as well as their origins [4, 5]. Multiple lines of evidence point to multiple potential introductions into the state. Populations in Southern California are thought to have arrived from Mexico, while populations in the Central Valley may have been introduced, in part, from the Southeastern United States. Upon detection in 2013, the Consolidated Mosquito Abatement District (CMAD) implemented an integrated vector control management strategy which involved extensive public education, thorough property inspections, sanitation, insecticide treatment at larval sources and residual barrier spraying with pyrethroids. Despite these efforts, *Ae. aegypti* successfully overwintered and continued to spread. The peridomestic habits of this mosquito make its reduction and eradication difficult. It is likely that the invasive populations of Ae. aegypti arrived in California with genetic mutations conferring resistance to the type I pyrethroid insecticides applied for vector control in California [6, 7].

Pyrethroid based compounds are used as adulticides and are favored for their efficacy and low mammalian toxicity [8]. Pyrethroids act on insect voltage gated sodium channels (VGSC) by binding to open channels and blocking the channel in the open conformation. This results in prolonged depolarization of the membrane and failure of neuronal function [9]. This class of insecticides is partitioned into two types (I and II) based on the presence or absence of a cyano moiety at the alpha carbon [10]. Point mutations within the channel domain of the protein can confer type-specific resistance by causing structural changes that reduce or eliminate the insecticides ability to bind the channel [11]. These mutations change the amino acid composition of the protein at specific locations that result in changes in charge and steric hindrance of the ion channel. These changes allow the VGSC to maintain normal function in the presence of pyrethroids.

**Fig. 1:**
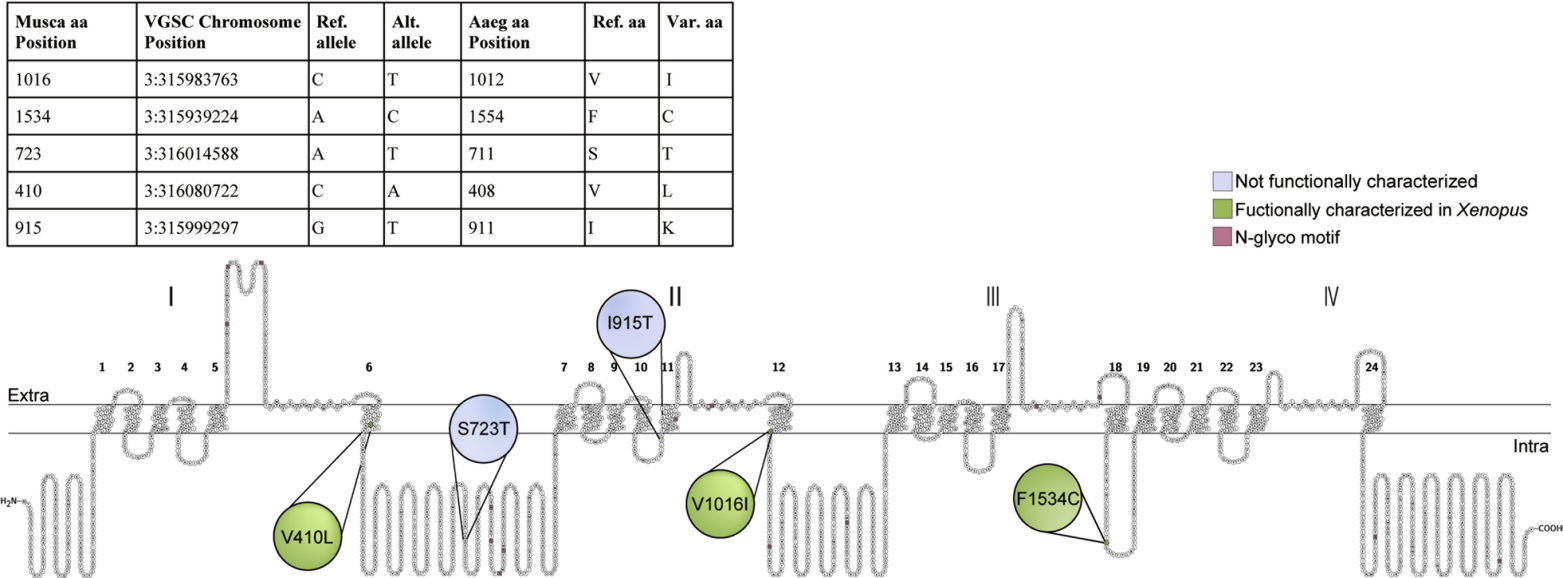
Topology of the mosquito sodium channel. The *Ae. aegypti* reference sequence was translated in CLC Main Workbench, Version 7. The resulting amino acid sequence was aligned to the *Musca domestica* (ANW06229) and *Drosophila melanogaster* (AAB59195) reference protein sequences. SNP annotations were transferred to *Musca* to determine the *Musca* protein position, and structural annotations were transferred from *Drosophila* to *Aedes*. The topology of the sodium channel was illustrated using Protter version 1 [49]. The sodium channel protein contains four homologous repeat domains (I–IV). Each repeat domain has six α-helical transmembrane segments (1–6, 7–12, 13–18, 19–24). Filled circles represent the five SNPs assayed in this study. Ref. = Reference. Alt. = alternate. aa = amino acid

Phenotypic analysis by CDC bottle bio-assay revealed that California *Ae. aegypti* were resistant to type I pyrethroids. This prompted researchers to determine the genomic sequence of the VGSC gene from representatives of populations from the Central Valley of California [6]. This sequencing revealed mutations known to be prevalent in *Ae. aegypti* from the Americas. Public health agencies began to screen for known pyrethroid resistance associated single nucleotide polymorphisms (SNPs) in the VGSC gene: F1534C, V1016I, and V410L [7]. These mutations are annotated based on their orthologous position in the *Musca domestica* VGSC protein (Genbank accession number: ANW06229) [12, 13]. V410L is located in the sixth transmembrane region of the first domain, V1016I is located in the sixth transmembrane region of the second domain, and F1534C is located in the sixth transmembrane region of the third domain (Fig. 1) [11, 14]. Recently, another mutation (S723T) was linked to Deltamethrin resistance, though its impact on the resistance phenotype remains unknown [15]. This SNP is localized in the intracellular region of the second transmembrane repeat domain. The F1534C mutation confers a low level of resistance on its own to type I pyrethroids. The V410L SNP, first described in 2017 [14], confers resistance to both type I and type II pyrethroids. The V1016I mutation on its own does not confer resistance, however in conjunction with F1534C it provides elevated insensitivity to type I and type II pyrethroids [9, 16, 17]. Mosquitoes homozygous for V1016I, F1534C, and V410L mutations exhibit a high level of resistance to both type I and type II pyrethroids [18]. As each SNP provides differing levels of protection against different classes of pyrethroids, testing for multiple SNPs in the field is relevant to screening for pyrethroid resistance [14].

Detecting and quantifying insecticide resistance in mosquitoes provides mosquito abatement groups with the tools to tailor their control strategies. The two primary methods of measuring resistance in the field are PCR testing for specific resistance associated alleles, and observing phenotypic resistance through the CDC Bottle Bioassay [19]. The CDC Bottle bioassay involves placing 10-25 mosquitoes in an insecticide coated Wheaton bottle followed by observation of the time and proportion of knockdown for 2 h after exposure. This procedure allows districts to investigate and quantify population level resistance. However, a direct analysis of the relationship between VGSC genotypes of individual mosquitoes and their associated bottle bioassay phenotypes (knockdown time) has not been completed with California *Ae. aegypti* populations. To measure the relationship between genotype and phenotype, individual mosquitoes were subjected to phenotypic assays followed by VGSC SNP genotyping of individuals at the upper and lower ends of the phenotypic spectrum. The *Ae. aegypti* samples were derived from multiple locations in California to explore population-level differences in response to insecticide exposure and VGSC genotype. We hypothesized that individuals carrying more resistance associated alleles will exhibit longer knockdown times when exposed to a modified bottle bioassay designed for individual mosquitoes.

## Materials and methods

### Mosquito collection and colony maintenance

**Fig. 2:**
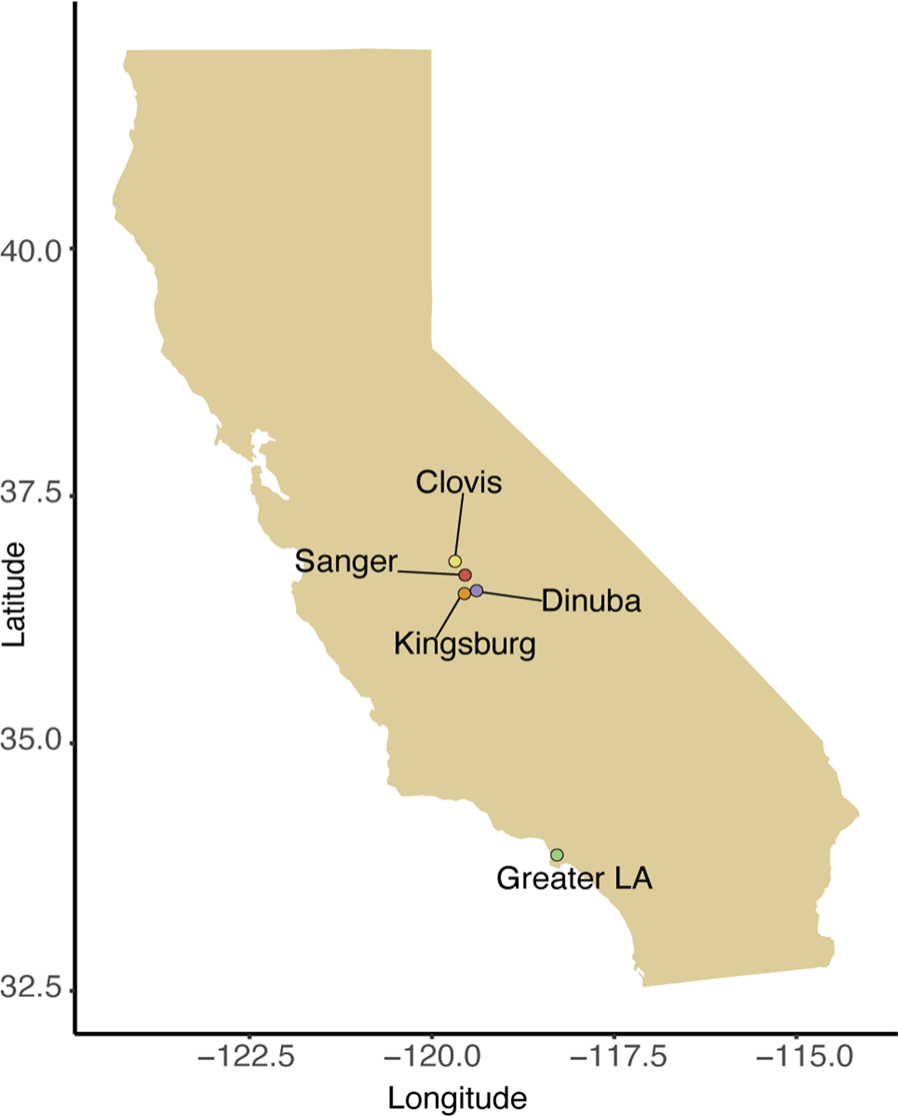
Locations from which *Ae. aegypti* were analyzed. Cities where founders for each lab strain were collected. Individuals were collected from the 5 cities we labeled on the map. Each lab strain was reared from individuals collected at various sites in these cities and reared together to increase specimen numbers.

Adult mosquitoes were collected from four towns in 2018 in the Central Valley of California: Dinuba, Clovis, Sanger and Kingsburg (Fig. 2) as well as from the Greater LA area. These sites were chosen because they have high prevalence of *Ae. aegypti,* and the inclusion of mosquitoes from both Southern California and the Central Valley encompass two genetically distinct groups. Eggs from these adults were collected, allowed to develop for at least 5 days, then flooded in trays of 1 l of water and reared according to standard protocols [20]. Mosquitoes were reared on a diet of ground rodent chow at 27 °C under 14:10 h (light:dark) photoperiod and adults were held at 70% relative humidity. Adults 1–3 days post-eclosion were then collected and individually exposed to a bottle bioassay to record individual knockdown time. Adult mosquitoes were fed on 10% sucrose solution *ad libitum* and did not receive a blood meal prior to insecticide exposure.

### Individual adult bottle bioassay

To determine time to knockdown for individual mosquitoes, a modified bottle bioassay was developed based on the CDC protocol [19]. 250 ml Wheaton bottles (Fisher #06-404B) were coated with technical grade pyrethrum purchased from Chem Service (West Chester, PA). Pyrethrum was chosen because it is the natural derivative of the pyrethroid class of insecticides and is used in California areas as adulticide. The pyrethrum (Lot #7581300) was diluted in acetone to a concentration of 15.6 μg/ml, the diagnostic dose recommended by the CDC for Aedes mosquitoes and bottles were coated with the insecticide following the procedure described in [21]. Individual female mosquitoes were then aspirated into each bottle and observed for knockdown for up to 2 h. Individuals were determined as knocked down when the bottle was rotated, and the mosquito could not reorient itself upright. Bottles were monitored continuously and exact time to knockdown was recorded. A susceptible lab colony, Rockefeller, was assayed as a reference for knockdown behavior in the bottle assay. For each population 80–95 adult female mosquitoes were assayed. Following knockdown, individuals were placed in the Lysis Buffer provided with the Zymo Quick DNA/RNA Miniprep kit (Cat #: D7001) and homogenized using Axygen mortar and pestles (Product PES-15-B-S1). Samples were stored at 4 °C.

### DNA extraction

DNA was extracted using the Zymo Quick-DNA/RNA Miniprep kit (Product D7001) using the suggested protocol for Solid Tissue Samples. DNA concentration for each sample was tested with a Qubit instrument (Life Technologies). Approximately 4 ng/µl in a total volume of 30 µl was extracted from each whole individual.

### SNP genotyping

**Fig. 3:**
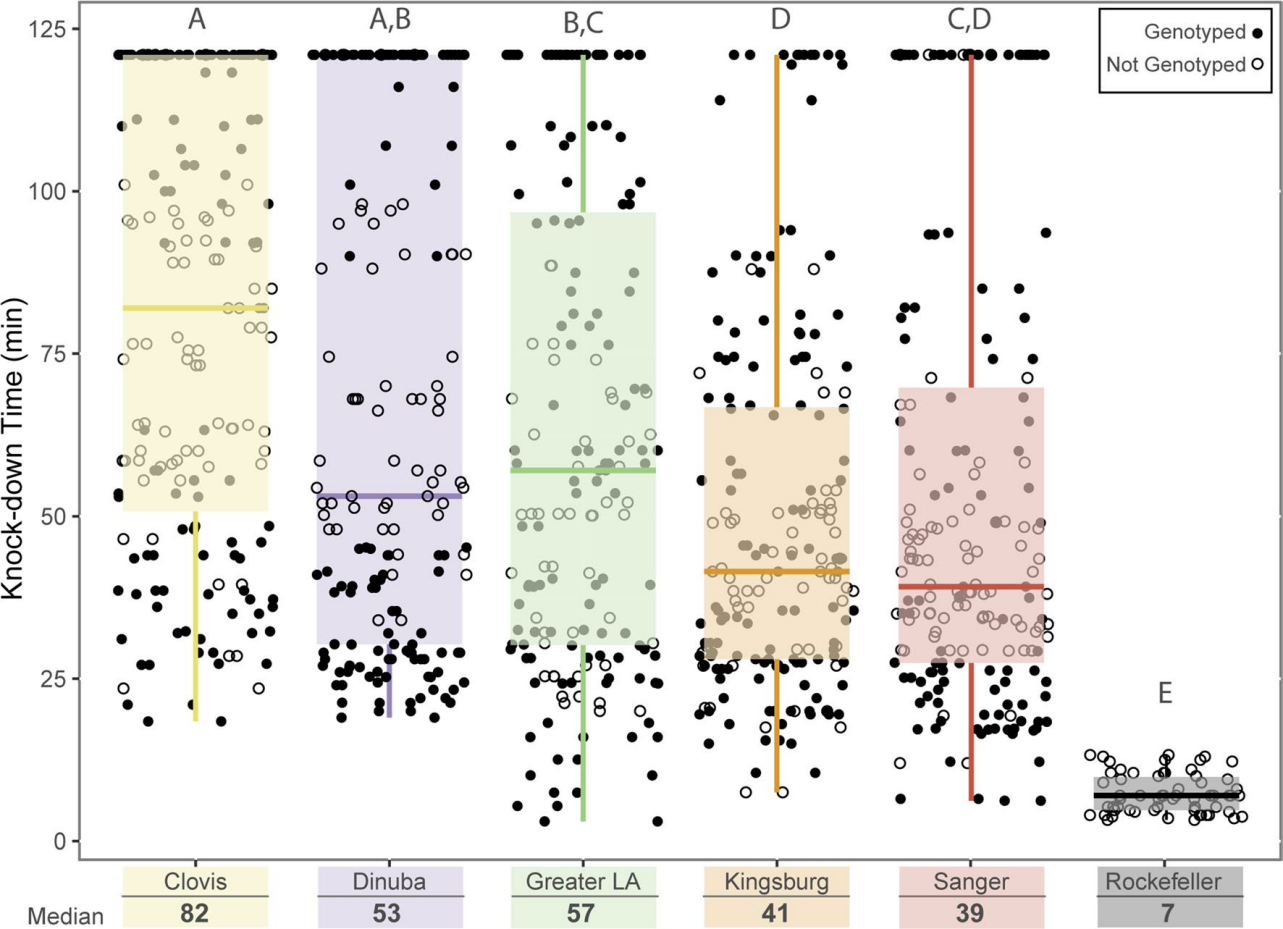
Distribution of knockdown times for each population. Each sample is represented by a circle. Black filled circles indicate genotyped samples, while the white circles represented samples that were not genotyped. Differences between group medians were determined by using the Mood’s median test followed by fdr correction. Letters indicate statistically significant differences.

A portion of bottle-assayed mosquitoes were sent for genotype analysis. Samples for genetic analysis were selected primarily from the upper and lower tertiles of knockdown times for each population (Table 1, Fig. 3). Our SNPs were identified from published whole genome sequences of *Ae. aegypti*. Sequences from the VGSC genomic locus of California mosquitoes were generated using primers reported in [22] (Genbank ID: KU728155-6). These sequences were aligned to the VGSC locus [AAEL023266 (3: 315,926,360–316,405,639)] in the *Ae. aegypti* AaegL5 reference genome*.* SNPs were identified within the coding regions of VGSC by comparison of aligned sequences against the reference sequence in VCF files using the Integrative Genomics Viewer [23]. Analysis of the data revealed 5 non-synonymous SNPs within the coding sequence. One additional mutation, previously reported from other studies [24], was included in case a limited sample size of genome data missed low frequency variations within the Californian derived sequences. The final 5 SNPs screened included 3:315939224 (F1534C), 3:315983763 (V1016I), 3:315999297 (T915K), 3:316014588 (S723T), and 3:316080722 (V410L). DNA was sent to the UC Davis Veterinary Genetics Laboratory for iPLEX assay using the MassARRAY System (Agena Biosciences, San Diego, CA) [25]. Detailed information on the 5 SNPs are provided in Additional file 1: Table S1.

**Table 1:** Resistance Allele frequency of the genotyped samples of Californian populations of *Aedes aegypti*

Genetic cluster	Population	N	F1534C				V1016I				V410L				S723T				I915K			
			SS	SR	RR	% R allele	SS	SR	RR	% R allele	SS	SR	RR	% R allele	SS	SR	RR	% R allele	SS	SR	RR	% R allele
Central Valley	Dinuba	60	0	0	60	100	0	3	57	97.50	0	3	57	97.50	0	3	57	97.50	0	3	57	97.50
	Clovis	60	0	1	59	99.17	0	1	59	99.17	0	9	51	92.50	0	9	51	92.50	0	0	60	100
	Kingsburg	64	0	0	64	100	0	0	64	100	0	0	64	100	0	0	64	100	0	0	64	100
	Sanger	58	0	0	58	100	0	0	58	100	0	0	58	100	0	0	58	100	0	0	58	100
Greater LA	Greater LA	67	0	1	66	99.25	11	37	19	59	11	37	19	59	11	37	19	59	0	15	52	88.80

### Data analysis

Statistical analysis was performed in R Version 3.5.2 [26]. Survival analysis was performed using the survival package [27]. The data was right-censored at 120 min, the end of the assay period. Pairwise comparisons of survival curves were performed through a log-rank analysis. Knockdown distribution normality was tested using the Shapiro–Wilk test in Base R. The box plot and histograms were created with ggplot2 [28]. *P*-value thresholds were adjusted for multiple comparison correction using the Benjamini and Hotchberg method [29] (Additional file 2: Table S2).

## Results

### Individual adult bottle bioassay

The knockdown times of all assayed mosquitoes are displayed in Fig. 3 and Additional file 3: Figure S1. Mosquitoes that did not knockdown during the assay period were coded as such and considered using the Kaplan–Meier analysis which accounts for right-censored data. Aside from the susceptible laboratory strain, Rockefeller, the Sanger and Kingsburg populations had the lowest median knockdown times and are significantly lower than that of Clovis or Greater LA (Fig. 3, Log-rank test < 0.05). The distribution of knockdown times for each of the five Californian populations sampled was non-parametric (Fig. 3, Additional file 3: Figure S1) and appear to potentially have bimodal distributions, though an assay with a greater dose of insecticide would be needed to confirm the bimodal nature of the distribution. The distribution reveals the presence of two subsets of mosquitoes in each population with one group succumbing to insecticidal effects around 35–45 min and the second group lasting up to and beyond the termination of the assay. However, the relative proportions of these distributions differ between populations (Additional file 3: Figure S1).

### SNP genotype by population

**Fig. 4:**
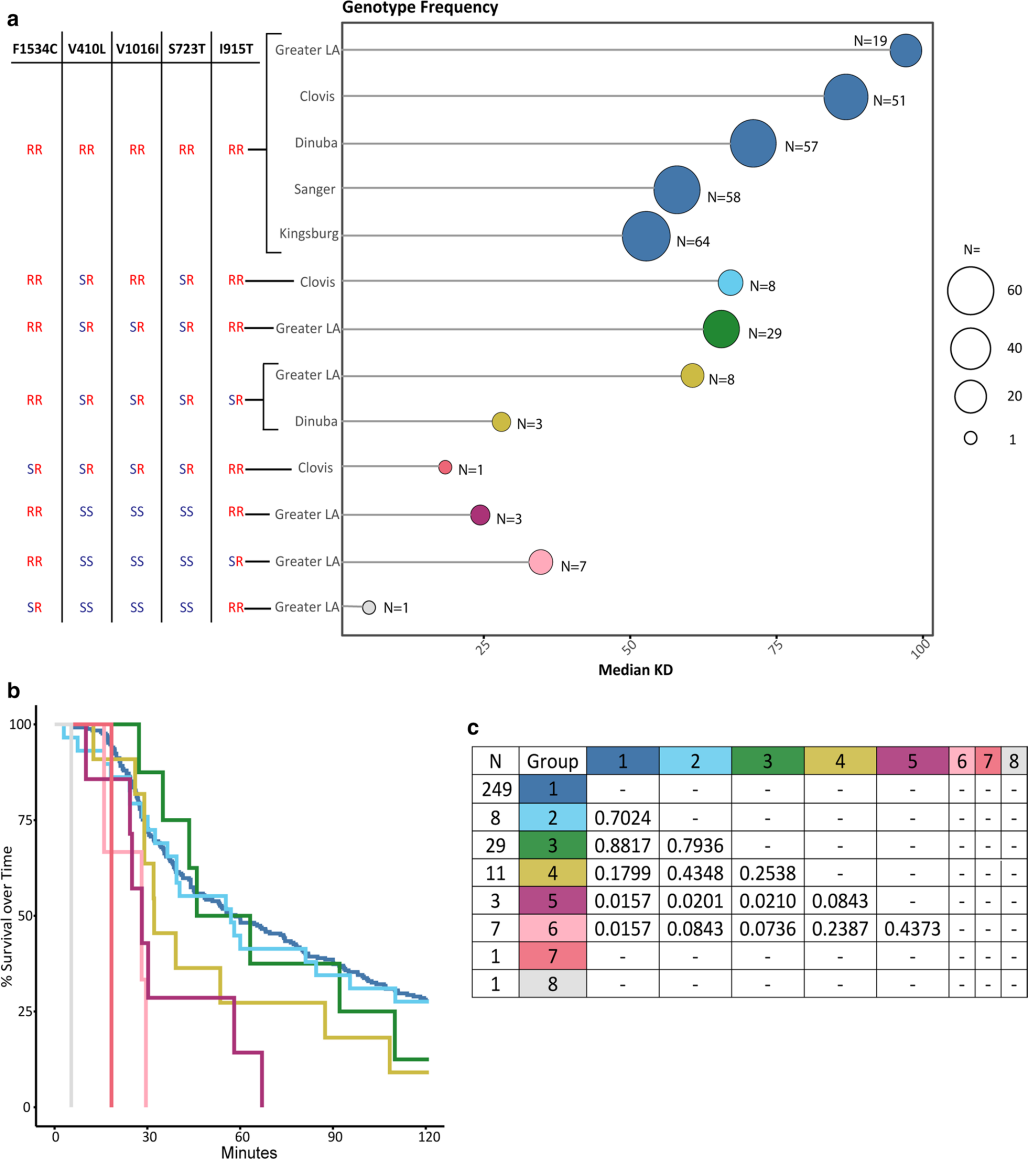
Median knockdown time for each present genotype. **a** Frequency of the 8 observed genotypes by population and their respective median knockdown times. **b** Kaplan-Meier analysis of observed knockdown between the 8 genotypes within all populations. C. *P*-values for log-rank comparisons between each genotype.

Samples for genetic analysis were selected primarily from the upper and lower tertiles of knockdown times for each population. We detected 5 SNPs within the sampled California populations (Table 1). All, except I915K, were reported previously [13, 14, 31]. The I915K SNP is in a region which is alternatively spliced and its presence in the resulting transcripts has not been determined conclusively. The assayed mosquitoes from the Central Valley populations (Clovis, Dinuba, Sanger and Kingsburg) are fairly homogenous with only 5% (12/242) of the mosquitoes tested showing heterozygosity at any of the 5 positions assayed (Fig. 4a). Alternatively, analysis of the Greater LA mosquitoes revealed that 71% had at least one susceptible allele at any of the five assayed SNPs (Table 1, 48/67). The F1534C mutation is nearly fixed across the genotyped subset of mosquitoes from all sampled populations. Only two mosquitoes were heterozygous (R/S) at position 1534. One individual from Clovis was heterozygotic at the amino acid positions F1534C, V1016I, V410L and S723T and one from LA was heterozygous at F1534C, and homozygous susceptible at V1016, V410 and S723 (Fig. 4a, Table 1). The I915K SNP was only found in a heterozygous conformation in the genotyped samples from the Dinuba population [5% (3/60)]. These mosquitoes were also heterozygous at the V1016I, V410L and S723T loci. In the Greater LA population 22% (15/67) were heterozygous at I915K. Of these 47% (7/15) were also homozygous susceptible at V1016, V410 and S723. The other 53% (8/15) were heterozygous at these positions (Fig. 4). The sites V1016I, V410L and S723T, are observed together frequently; an individual heterozygous or homozygous at one site was correspondingly heterozygous or homozygous across all three with exceptions in the Clovis population. In the Clovis population 13% (8/60) mosquitoes were heterozygous only at sites S723T and V410L (Fig. 4a).

### VGSC SNP genotype and knockdown time

The majority of the genotyped individuals were homozygous for the resistance associated alleles at all five loci tested. However, 0.8% (2/246) individuals with this genotype, both from Sanger, demonstrated knock down in as little as 6 min after exposure to pyrethrum, overlapping with the susceptible Rock strain. Of the individuals with all 5 alternate resistance associated alleles 9% (23/246) knocked down in 20 min or less. A higher level of diversity of VGSC genotypes (6) was observed in the Greater LA mosquitoes relative to the Central Valley populations (4) (Fig. 4a). This is consistent with previous reports showing little to no polymorphism in VGSC SNPs in central California populations [5, 7]. The increased genetic variation observed in the Greater LA population relative to the Central Valley population is also consistent with a previous study monitoring both Central Valley and southern California locations [7]. It is noteworthy that independent studies utilizing whole genome sequence [5], SNP chip [4], and microsatellite [4] indicated different genetic makeup separating Southern CA and Central CA. Of the mosquitoes tested from populations in the Central Valley, only 5% (3/60) and 15% (9/60) samples from Dinuba and Clovis respectively carried “susceptible” alleles (Fig. 4a). Of note, <1% (2/309) of the mosquitoes tested from all populations were heterozygous at position F1534C, and those mosquitoes knocked down at 5 (Greater LA) and 18 (Clovis) minutes, though it is difficult to ascertain the significance of this result with such a low frequency of this genotype.

### Survival analysis

**Fig. 5:**
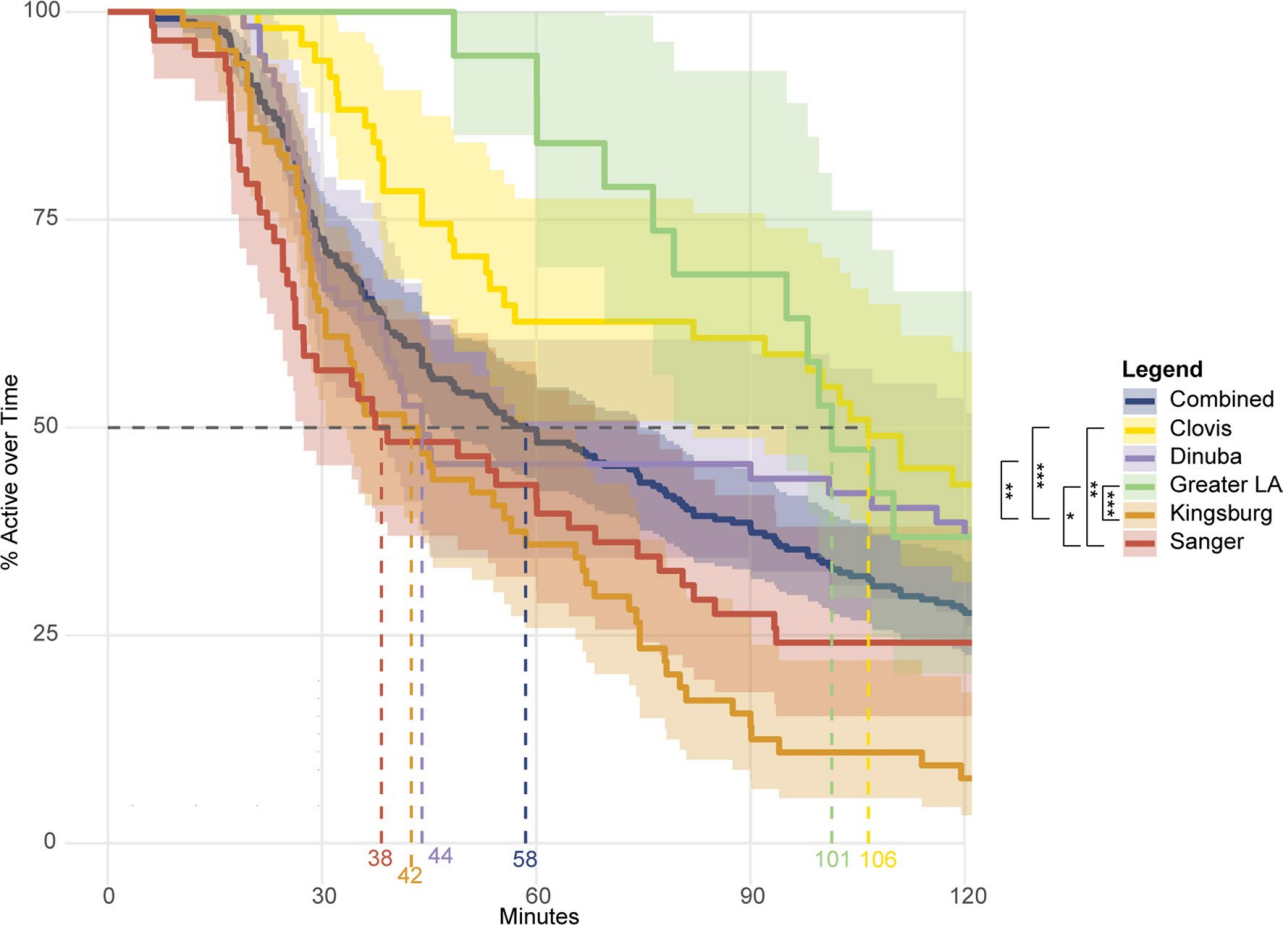
Kaplain-Meier survival curve analysis of the homozygous resistant genotype. A comparison of knockdown between the homozygous resistant phenotypes from all 5 populations tested. ***P < 0.001, **P < 0.01, *P < 0.05.

To compare the relationship between genotype and phenotype, Kaplan-Meier survival analysis was utilized to account for individuals that may not have experienced the “event” (in this case, knockdown) during the study period [30]. Survival curve analysis of mosquitoes with equivalent genotypes reveals a range of median knockdown times that roughly correlate with the relative proportions of susceptible versus resistant VGSC alleles. In cases where only a single individual represented a genotype, *P*-values were not reported (Fig. 4c). In general, individuals containing susceptible VGSC alleles did not last as long as those carrying the fully resistant genotype. However, a small proportion of individuals carrying the homozygous resistant genotype at all alleles have knockdown times equivalent to individuals with homozygous susceptible genotypes (Fig. 4a, b). We reason this is unlikely due to methodological error, as these low knockdown times were observed across multiple populations. When controlling for genotype, there were still significant differences between the strains (Fig. 5). All the Sanger and Kingsburg mosquitoes were homozygous at all 5 resistance loci and had median knockdown times of 38 min (*N* = 58) and 42 min (*N* = 64) respectively (Fig. 5). This observation contrasts with median knockdown times observed in mosquitoes from the Clovis (106 min) and Greater LA (101 min) populations with the identical VGSC genotype. Statistical comparison of the survival curves of these groups demonstrates significant differences in knockdown times between populations even though the individuals tested carry identical VGSC resistance loci.

## Discussion

Using the individual bottle bioassay in this study allowed us to explore the phenotypic variability of resistance in *Ae. aegypti*, and pair that phenotypic information with genotype, a previously unexplored relationship in *Ae. aegypti*. This individual bottle bioassay method yielded a non-normal, potentially bimodal distribution of knockdown times. In general, individuals with susceptible alleles succumbed to knockdown earlier, however, there was some overlap in the knockdown times of individuals with fully resistant genotypes and those with susceptible alleles.

Target site mutations in the VGSC gene were prevalent in our sample populations and play an important role in resistance. Yet, significant variability in average knockdown times was observed between populations when controlling for genotype. This observation suggests the presence of other mechanisms of resistance that vary in frequency between populations. Another documented mechanism of pyrethroid resistance in *Ae. aegypti* is metabolic detoxification by enzymes such as Cytochrome P450s and GSTs [31–33].

The observed variability between populations indicates that other factors are at play in mediating pyrethroid resistance in these mosquitoes and that these factors are not universally distributed within and between populations. The establishment of *Ae. aegypti* in California has raised concerns that they could facilitate local transmission of arboviruses such as dengue and Zika as seen in Florida, Texas and Puerto Rico [34–36]. While pyrethroids are favored as a chemical method for their control, resistance is widespread in California and globally [6, 7, 9, 37]. Despite significant work on pyrethroid resistance in *Ae. aegypti*, questions remain concerning the identification and relative compounding roles that VGSC mutations, metabolic detoxification mechanisms, other resistance mechanisms, and environmental factors have on conferring insecticide resistance. Additional resistance mechanisms such as reduced cuticular permeability and behavioral resistance have been shown to be significant in insects [38, 39].

The VGSC mutations V1016I, F1534C and V410L are prevalent in *Ae. aegypti* in the Americas and have been closely monitored by the California Department of Public Health (CDPH) [7, 14, 16, 18]. These surveillance efforts found that between 2015 and 2017 resistant alleles were largely fixed in the Central Valley population, and less abundant (> 80% for F1534C and > 61% for V1016I from 2015–2017) but increasing in prevalence in the Southern population of *Ae. aegypti* [7]. Studies in Mexico have identified similar trends [18]. Our results were similar to the CDPH findings (Table 1). In our samples F1534C was nearly fixed, with only two heterozygous individuals (one from Clovis and one from Greater LA). The detection of a heterozygote from Clovis was surprising, given our relatively small sample size compared to the 1200 mosquitoes tested by Liebmen et al. between 2015 and 2017. Our sample mosquitoes were captured in late 2018, and follow the general trend found in [7]. In California, *Ae. aegypti* appear to have dispersed along the major Interstate 5 route so it is possible that mosquitoes from the Southern Population and Central Valley population are moved between regions [3]. Recent whole genome sequencing data as well as SNP chip data support multiple introductions of *Ae. aegypti* into CA and found evidence of distinct genetic clusters converging in the Central Valley [4, 5].

The majority of the tested individuals from our Central Valley samples carried all 5 VGSC mutations (Figs. 4, 5). Resistance alleles were likely prevalent in the founder populations in California [6]. Resistance is widespread in populations in the United States, though the susceptible F1534 allele is still found in some areas [40, 41]. Interestingly, bottle bioassay results reported by local control agencies had not always followed the patterns that would be expected given the relative proportions of resistance associated SNPs, which was also in line with our results using the individual bottle bioassay test (Figs. 3, 4, 5). It will be an important next step to test the association between the SNPs identified in this study and Deltamethrin, a type II pyrethroid, as this ingredient is used more widely throughout the state.

The *Xenopus* oocyte expression system for *Ae. aegypti* sodium channels has facilitated investigation into the role of individual mutations assayed in pyrethroid resistance [14, 42–44]. The mutations V410L + F1534C and V1016I + F1534C have even been studied in combination [14, 17, 44]. However, little is known about the S723T mutation [45] though with the high frequency of this mutation in all populations tested (59% in Greater LA and > 92% in all Central Valley populations, Table 1), future functional analyses would be important to understanding the role this mutation plays in resistance. These mutations act in combination with metabolic mechanisms of pyrethroid resistance mediated by the upregulation, overexpression or duplication of cytochrome P450 enzymes encoding genes [15, 46–48]. Bottle bioassays with cytochrome P450 inhibitor, piperonyl butoxide (PBO), have implicated that cytochrome P450s play a synergistic role in combination with VGSC mutations to confer resistance in mosquitoes from Clovis [6]. The significant differences in knockdown time found in our samples with the resistant genotypes (Fig. 5) supports the hypothesis that other mechanisms are important and variable within California populations and these other mechanisms should be explored in future studies. This indicates that running bottle bioassays that include the cytochrome P450 inhibitor PBO will provide local control agencies important information when evaluating resistance. Given the functional evidence in the literature, and our assay results (Figs. 4, 5), the increasing prevalence of the F1534C mutation, particularly in combination with V410L and V1016I reliably indicates an elevated level of resistance to type I pyrethroids [7, 14, 42, 44]. However, the individual bottle assays employed here prompt us to question that, assuming good conditions, the presence of resistance associated alleles in the VGSC gene may not guarantee phenotypic resistance. With this in mind, it is important to carefully explore the role of specific alleles on resistance to further inform control efforts. For this reason, it is important to pursue detailed investigations in the biology underlying the resistance phenotype. Additional knowledge on this subject would facilitate identification of additional genetic and/or biochemical markers. The ideal goal would be to identify markers that provide quantitative measures of the resistance phenotype in field caught mosquitoes which would boost the predictive power of these assays. In addition, identification of new targets underlying the phenotype opens the door for development of alternative strategies and new synergists to augment existing insecticidal compounds.

## Supplementary Information

**Additional file 1: Table S1.** iPLEX MassARRAY primers for 5 SNPs used in this study.

**Additional file 2: Table S2.** Population name and generation for bottle assay testing.

**Additional file 3: Figure S1.** Histogram of knockdown times for assayed mosquitoes with kernel density plot. Knockdown time distribution was non-normal (Shapiro-Wilk test, p <0.00005 for each population).

## Data Availability

The datasets used and/or analyzed during the current study are available from the corresponding author on reasonable request.
