# Supplementary material for: Frequency of sodium channel genotypes and association with pyrethrum knockdown time in populations of Californian Aedes aegypti
Source: Parasit Vectors. 2021 Mar 6;14:141. doi: 10.1186/s13071-021-04627-3 (PMC7936502; doi:10.1186/s13071-021-04627-3)
Supplement: Supplementary file 2 — Additional file 2: Table S2. Population name and generation for bottle assay testing. [file 13071_2021_4627_MOESM2_ESM.docx]

Table. S2 Population name and generation for bottle assay testing

| Sample origin | Generation | Assayed/Collected |
| --- | --- | --- |
| Greater Los Angeles (Los Angeles County) | F2 | 67/91 |
| Sanger (Fresno County) | F1 | 58/103 |
| Kingsburg (Fresno County) | F1 | 64/99 |
| Dinuba (Tulare County) | F3 | 60/88 |
| Clovis (Fresno County) | F2 | 60/95 |
