# Supplementary figures and images for: Frequency of sodium channel genotypes and association with pyrethrum knockdown time in populations of Californian Aedes aegypti
Source: Parasit Vectors. 2021 Mar 6;14:141. doi: 10.1186/s13071-021-04627-3 (PMC7936502; doi:10.1186/s13071-021-04627-3)

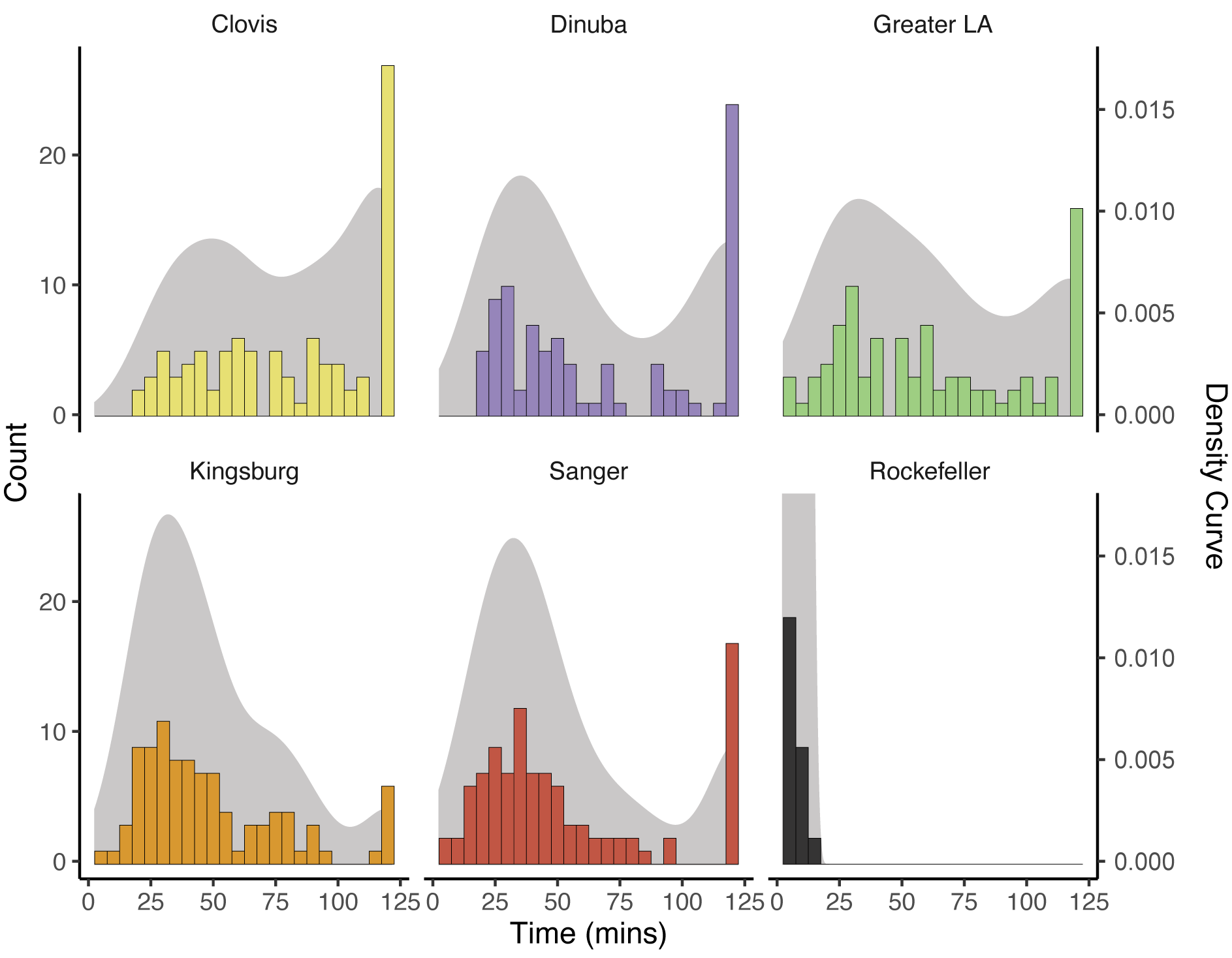

Supplement: Supplementary file 3 — Additional file 3: Figure S1. Histogram of knockdown times for assayed mosquitoes with kernel density plot. Knockdown time distribution was non-normal (Shapiro-Wilk test, p <0.00005 for each population). [file 13071_2021_4627_MOESM3_ESM.tif]
